# Identification of Three New *N*-Demethylated and *O*-Demethyled Bisbenzylisoquinoline Alkaloid Metabolites of Isoliensinine from Dog Hepatic Microsomes

**DOI:** 10.3390/molecules171011712

**Published:** 2012-10-01

**Authors:** Hui Zhou, Liping Li, Huidi Jiang, Su Zeng

**Affiliations:** Department of Pharmaceutical Analysis and Drug Metabolism, College of Pharmaceutical Sciences, Zhejiang University, Hangzhou 310058, Zhejiang, China

**Keywords:** bisbenzylisoquinoline alkaloids, demethylation, HPLC, isoliensinine, microsomes, tandem mass spectrometry

## Abstract

Isoliensinine, a natural phenolic bisbenzyltetrahydroisoquinoline alkaloid, has received considerable attention for its potential biological effects such as antioxidant and anti-HIV activities. From the dog hepatic microsomes of isoliensinine, three new *N*-demethylated and *O*-demethylated metabolites, 2-*N*-desmethyl-isoliensinine (**M1)**, 2'-*N*-desmethylisoliensinine (**M2**), and 2'-*N*-6-*O*-didesmethylisoliensinine (**M3**), were identified by high-performance liquid chromatography and data-dependent electrospray ionization tandem mass spectrometry. Possible metabolic pathways for isoliensinine have been proposed. The result should prove very helpful for evaluation of the drug-like properties of isoliensinine and other bisbenzylisoquinoline alkaloids.

## 1. Introduction

Bisbenzylisoquinoline alkaloids are among the most important and widely distributed alkaloids in plant resources [1,2]. There are several types of bisbenzylisoquinoline alkaloids, including one, two, or three diphenyl ether linkages and other phenyl ether linkages [2]. Isoliensinine and its analogues liensinine and neferine are three tail-to-head phenolic benzyltetrahydroisoquinoline alkaloid dimers, found to be the major alkaloids in the lotus (*Nelumbo nucifera* GAERTNER., Nelumbonaceae), especially in its embryo loti “Lien Tze Hsin” (green embryo of mature seed), which is a famous Traditional Chinese Medicine (TCM), primarily used for nervous disorders, insomnia, high fevers with restlessness, and cardiovascular diseases [3,4,5], and also used as an antifebrile, sedative and hemostatic agent [6]. In recent years, these bisbenzylisoquinoline alkaloids were found to have wide biological activities such as antioxidant action, cardiovascular pharmacological effects such as antihypertensive and antiarrhythmic actions [7,8,9], reversing the multidrug resistance (MDR) effect of human carcinomas [10,11], anti-HIV activity [12], and as anti-tuberculosis agents toward multidrug-resistant *Mycobacterium tuberculosis* [13]. In addition, isoliensinine can inhibit bleomycin-induced pulmonary fibrosis in mice [14] and decrease the overexpression of growth factors PDGF-beta, bFGF, proto-oncogene c-fos, c-myc and hsp70 [15].

In spite of the growing interest in the biological properties of isoliensinine and its analogues, most studies are focused on their isolation and pharmacology, and few studies have investigated their pharmacokinetics [16,17,18] and metabolism [19,20]. In fact, determination and characterization of their metabolites and metabolic pathways are very important for drug discovery and development. Our previous study [21] revealed the fragmentation patterns of isoliensinine and its analogues, and also identified six metabolites of neferine. However, to our knowledge, to date there are no details on the metabolite of isoliensinine.

In this paper, we report three novel metabolites of isoliensinine obtained from dog hepatic microsomal incubations and identified using reversed-phase high performance liquid chromatography (RP-HPLC) and electrospray ionization tandem mass spectrometry (ESI-MS/MS). As a result, these three metabolites were found to be novel desmethyl or didesmethyl products of isoliensinine. The possible metabolic pathways for isoliensinine are proposed. To the best of our knowledge, this is first report of these microsomal metabolites and the metabolic pathways of isoliensinine. It should prove very helpful for evaluation of the drug-like properties of isoliensinine and other bisbenzylisoquinoline alkaloids.

## 2. Results and Discussion

**Figure 1:**
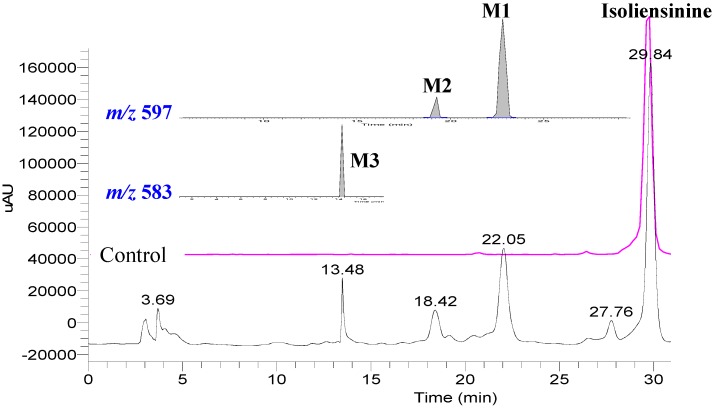
HPLC-UV-ESI-MS profiles of the microsomal metabolites of isoliensine. The UV chromatogram was recorded at 280 nm, and selected ion chromatograms were pre-set at *m/z* 583 and 597 using data-dependent mode. Pink line shows the chromatogram of the control sample in the absence of NADPH.

The hepatic microsomes were first prepared according to a reported process [21], and then incubated *in vitro* with 100 μM isoliensinine. After transformation, an organic solvent was used to extract metabolites which were then analyzed by RP-HPLC. In order to find more bisbenzylisoquinoline metabolites, an on-line DAD detector was set at 200–400 nm to record the UV spectra. As shown in Figure 1, isoliensinine was found to be metabolized into three major metabolites, identified as **M1**, **M2** and **M3**, and several minor metabolites. Here we focus on characterizing the structures of the three major metabolites.

**Figure 2:**
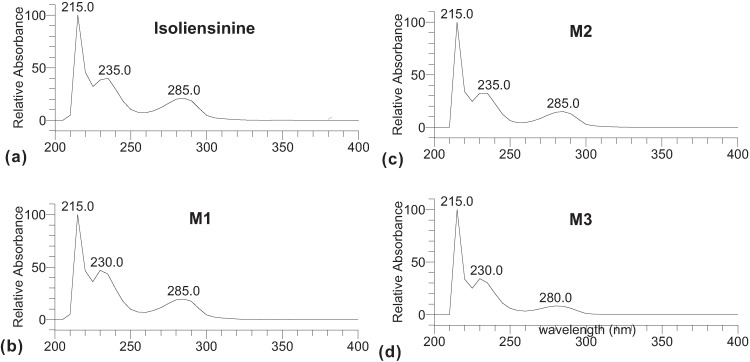
DAD spectra of (**a**) standard isoliensinine and (**b**–**d**) its microsomal metabolites **M1**–**3**.

The on-line DAD spectra (Figure 2) showed the three metabolites have very similar UV spectra to their parent compound isoliensinine and maximum absorbance at 215, 230, and 285 nm, implying that they possess the very similar molecular structures to their parent compounds.

A previous study [21] revealed that demethylation is the major metabolic path *in vitro*. In order to identify the metabolites, the data-dependent product MS/MS scans were performed for detection of the pre-listed compounds. As shown in Figure 1, metabolites **M1** and **M2** were found to have the same protonated molecular ion [M+H]^+^ at *m/z* 597, implying that they are produced by loss of a methyl group from isoliensinine, and metabolite **M3** possess a [M+H]^+^ at *m/z* 583, corresponding to loss of two methyl groups.

**Figure 3:**
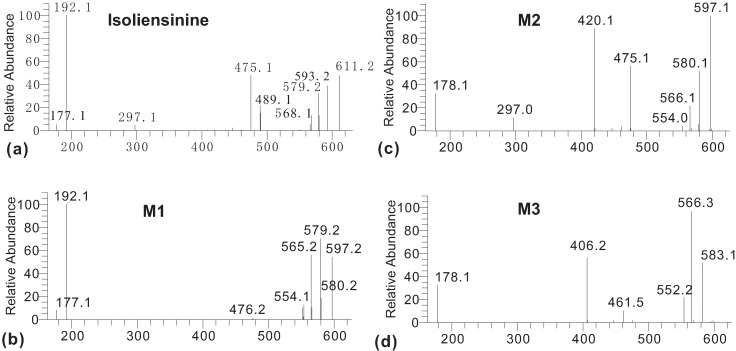
Positive ESI-MS/MS spectra of (**a**) standard isoliensinine and (**b**–**d**) microsomal metabolites **M1**–**3**.

**Scheme 1:**
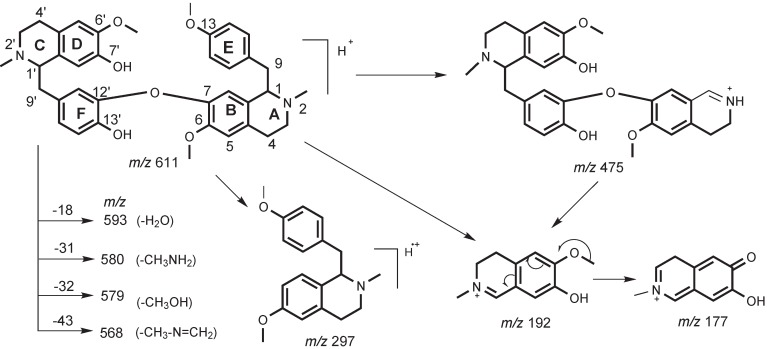
Proposed fragmentation patterns of isoliensinine.

Further ESI-MS/MS analyses (Figure 3) showed that these metabolites have typical bisbenzylisoquinoline characteristics. As reported [21], the ESI-MS/MS spectrum of isoliensinine clearly shows the major diagnostic fragment ions at *m/z* 192, 475, 489, 593, 580 and 568 resulting from the cleavage of the C1'-C9' bond resulting in positive groups CD (Scheme 1), and the loss of 4-ethyl-1-phenol or 4-ethyl-1-methoxybenzene following rearrangements [21,22,23]. In addition, H/D exchange ESI-MS/MS spectra [21] have showed the relative stable fragmentation ions formed by the elimination of H_2_O, CH_3_NH_2_, CH_3_OH, and CH_3_–N=CH_2_. Thus, a possible fragment pattern of isoliensinine as shown in Scheme 1 can be proposed. Clearly, the ions such as at *m/z* 611, 475 and 192 were key fragment ions to identify its metabolites from dog hepatic microsomes.

The ESI-MS^2^ of the metabolite **M1** (Figure 3b) displays characteristic fragmentation ions at *m/z* 597, 580, 579, 565, 192 and 177. The existence of the diagnostic fragment ion at *m/z* 580 corresponding to the loss of NH_3_[24] indicated **M1** is a *N*-desmethyl metabolite of isoliensinine. There are two possible positions to lose an *N*-methyl group, one is the 2-*N*-methyl group, and the other is the 2'-*N*-methyl group. The prominent fragments ions at *m/z* 192 and 177 indicated that metabolite **M1** has the same CD group as its parent isoliensinine, without the loss of the 2'-*N*-methyl group. Therefore, the lost group is the 2-*N*-methyl group, which is further supported by the absence of characteristic ions at *m/z* 475 and 297, corresponding to the ABFCD or ABE (Scheme 1) moieties, respectively. Due to the loss of the 2-*N*-methyl group, the ABFCD (Scheme 1) moiety of the metabolite **M1** showed a weak capacity to form positive ions at *m/z* 475. Based on these evidences, the metabolite **M1 **was identified as 2-*N*-desmethylisoliensinine.

**Scheme 2:**
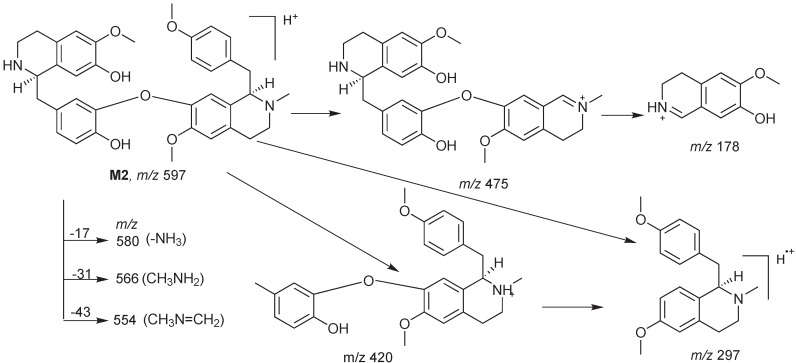
Proposed fragmentation of metabolite M2.

Metabolite **M2** is also an *N*-demethylation product of isoliensinine because its ESI-MS^2^ spectrum (Figure 3c) showed the prominent diagnostic fragment ion at *m/z* 580 corresponding to the loss of NH_3_ [24]. However, differing from the metabolite **M1**, the loss of the *N*-methyl group of **M2** occurred on the 2'-*N*-methyl group of the CD moiety because of the existence of the characteristic fragment at *m/z* 178. Furthermore, the prominent characteristic fragments ions at *m/z* 420 and 297, as well as the ion at *m/z* 475, were supportive of the above elucidation. Its fragmentation patterns were illustrated in Scheme 2. Based on these data, metabolite **M2** can be identified as 2'-*N*-desmethylisoliensinine. 

Metabolite **M3** showed the protonated ion [M+H]^+^ at *m/z* 583 (Figure 1), implying that it is probably produced by the loss of two methyl groups. Its ESI-MS/MS spectrum (Figure 3d) showed similar fragment profiles to metabolite **M2**, implying structural similarity. The diagnostic fragment ion at *m/z* 566 corresponding to the loss of NH_3_, indicated that the metabolite **M3** is an *N*-desmethyl product. The presence of a characteristic fragment at *m/z* 178 indicates that one lost group occurred on the 2'-*N*-methyl group of the CD moiety. Furthermore, another demethylation can determined at the position of the 6-*O*-methyl group of the AB moiety due to the presence of the intensive fragment ions at *m/z* 461 and 406. Based on these data, the metabolite **M3** can be unambiguously identified as 2'-*N*-6-*O*-didesmethylisoliensinine.

**Scheme 3:**
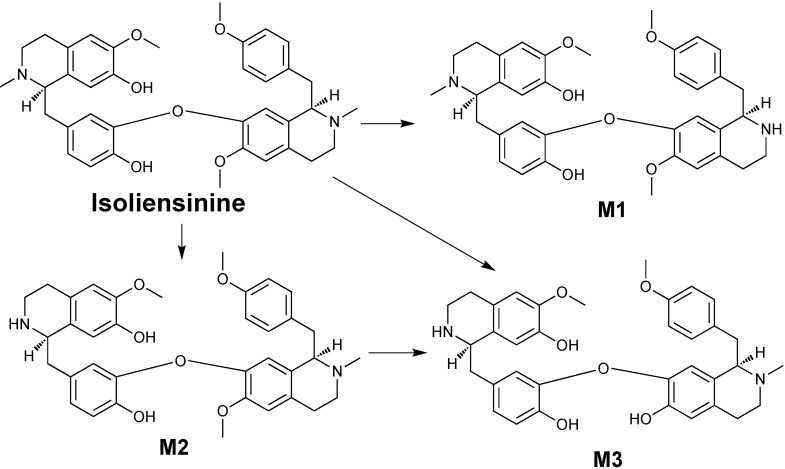
Proposed metabolic pathway of isoliensinine in dog hepatic microsomes.

The occurrence of these new metabolites provides evidential information about the biotransformation of bisbenzylisoquinoline alkaloids. A recent study [25] assumed that in *N. nucifera* isoliensinine may be biosynthesized from two molecules of (*R*)-*N*-methylcoclaurine via C–O oxidative coupling and subsequent *O*-methylation. In addition, isoliensinine may transform into neferine by further *O*-methylation [25]. Interestingly, the biotransformation in dog hepatic microsomes is a reversed metabolic process comprising biosynthesis of these bisbenzylisoquinoline alkaloids in *N. nucifera*. Our previous study indicated that neferine could be metabolized into isoliensinine [21]. The present study indicated that isoliensinine can be further metabolized into three major bisbenzyl-isoquinoline alkaloids **M1**, **M2**, **M3**. The possible metabolic pathway of isoliensinine was summarized in Scheme 3. Clearly, *N*-demethylation and *O*-demethylation are two important metabolic pathways of isoliensinine in dog hepatic microsomal incubations, as reported previously for neferine [21].

Demethylation is an important metabolic dealkylation process for compounds with alkyl groups [26]. In the rat hepatic S9 fraction in the presence of an NADPH-generating system, *N*-demethylation was one of the metabolic pathways of thalicarpine [24] and dauricine [27], which are tail-to-tail benzyltetrahydroisoquinoline alkaloid dimers. A recent study [20] has suggested that both CYP3A and CYP2B are involved in the metabolism of neferine in rat liver microsomes, and CYP3A probably plays a major role. Another study indicated CYP2D6 is also involved in the liver metabolization of neferine to liensinine, isoliensinine and other demethylated metabolites [19]. In the present study, three desmethyl metabolites of isoliensinine are also possibly produced by catalysis by the cytochrome P450 enzyme systems of dog hepatic microsomes.

## 3. Experimental

### 3.1. Chemicals and Reagents

DL-Isocitric acid, isocitric dehydrogenase, β-nicotinamide adenine dinucleotide phosphate reduced form (β-NADPH), and β-nicotinamide adenine dinucleotide phosphate (β-NADP) were purchased from Sigma Chemical (St. Louis, MO, USA). Acetonitrile and methanol used for HPLC analyses were purchased from Tedia Company (Fairfield, TX, USA). Deionized water was purified using a Milli-Q system (Millipore, Milford, MA, USA). The bisbenzylisoquinoline alkaloid isoliensinine used for authentic standards of more than 98% purity were isolated from seed embryos of *N. nucifera* GAERTN according to the reported counter-current chromatography (CCC) process [28]. All other chemicals and solvents were of analytical grade.

### 3.2. Preparation of *in Vitro* Microsomal Metabolites of Isoliensinine

*In vitro* microsomal incubation was performed as reported [21]. In short, dog hepatic microsomes were first prepared according to the method of Gibson and Skett [29] using beagle dogs (each 13.0–15.0 kg, Experimental Animal Center of Zhaoqin, Guangdong Province, China). Then, microsomal incubation mixture (1 mL) composed of 0.1 M (pH 7.4) Tris-HCl buffer, 15 mM MgCl_2_, 10 mM DL-isocitric acid, 0.8 unit/mL isocitric dehydrogenase, 0.5 mg/mL microsomal protein and 100 µM isoliensinine was pre-incubated at 37 °C for 5 min. The reaction was initiated by adding NADP/β-NADPH (0.6 mM/0.3 mM). After incubation at 37 °C for 120 min, the reaction was terminated by the addition of ice-cold ether (3 mL) and NH_3_-NH_4_Cl buffer (1 mL, pH 10), the mixture was centrifuged at 3,000 *g* for 5 min. The supernatant was evaporated to dryness under nitrogen, the residue was dissolved in mobile phase (200 µL) before HPLC-ESI-MS/MS analysis. The blank controls were carried out without β-NADPH and terminated directly, then prepared the same as the above samples.

### 3.3. Characterization of Metabolites by HPLC-ESI-MS/MS with Data-Dependent Mode

HPLC-ESI-MS^n^ analyses were performed on a Surveyor HPLC system (Thermo Electron, San Jose, CA, USA) coupled with a Finnigan MAT LCQ ion trap mass spectrometer (ThermoQuest-Finnigan Co., San Jose, CA, USA). The HPLC column used is an Agilent Zorbax Extend C18 (250 mm × 4.6 mm i.d., 5 µm, Agilent Technilogies Inc., Santa Clara, CA, USA) with a C18 guard column (10 mm × 5 mm i.d.). In order to avoid peak tailing of the alkaloid metabolites on the column, basic triethylamine was added to form a 0.1% aqueous solution (Solvent A). A gradient separation at 0.5 mL/min at 25 °C was set: 0–35 min, solvent A from 70% to 40% and solvent B (acetonitrile) from 30% to 60%, and then maintaining 40% solvent A and 60% solvent B to 40 min.

After HPLC column separation, the effluent from the DAD detector was directed into the ion source of the MS instrument. There was a short delay of about 0.2 min between the DAD and MS detectors because of the connecting tubing between the HPLC and MS instruments. The ESI-MS/MS conditions were configured as follows: spray voltage, 4.5 kV; capillary voltage, 10 V; capillary temperature, 270 °C; sheath gas, nitrogen setting at 80 units; auxiliary gas, nitrogen setting at 20 units; collision gas, helium used as for the tandem mass experiments; scanning modes: full scan ranging from *m/z* 150–1,000 Da for all metabolites and selection ion scan at *m/z* 597, and 583 for demethylated metabolites. The isolation width was set to 1 Da, and ejected ions were detected with the electron multiplier set at a gain of 5 × 10^5^. The Xcalibur software version 1.0 was used to acquire and process the MS data. 

## 4. Conclusions

This work identified three novel metabolites of isoliensinine obtained from dog hepatic microsomes. These metabolites could be formed by demethylation or bisdemethylation of the parent isoliensinine. They showed diagnostic MS fragments such as elimination of benzyl group E or F, and losses of H_2_O, CH_3_NH_2_, CH_3_OH, and CH_3_–N=CH_2_. To the best of our knowledge, this is first report of the microsomal metabolites and the metabolic pathway of isoliensinine. The liver is a very important organ for drug metabolism. Although the current work only focused on the *in vitro* microsomal metabolism of isoliensinine, the metabolism and the metabolites are very important for further pharmacokinetic and pharmacodynamic studies of isoliensinine and other bisbenzylisoquinoline alkaloids. It is also important for screening and developing new phenolic bisbenzyltetrahydroisoquinoline alkaloids for clinical therapy.
